# Organizational and community determinants of hospital-based violence prevention programs in the United States

**DOI:** 10.1016/j.puhip.2026.100794

**Published:** 2026-05-07

**Authors:** Aizhan Karabukayeva, Halley Reeves, Reena Joseph Kelly, Neeraj Puro

**Affiliations:** a Department of Health Administration and Policy, Hudson College of Public Health, University of Oklahoma Health Sciences, United States; b Department of Population Health & Leadership, University of New Haven, United States; c Management Programs-Health Administration, Florida Atlantic University, United States

## Abstract

**Background:**

Hospital-based workplace violence prevention programs (WVPPs) and community violence prevention programs (CVPPs) are increasingly used to protect healthcare workers and respond to violence-related harm. National evidence on which hospitals report these programs remains limited. This study examined national patterns of annual WVPP and CVPP reporting in U.S. hospitals and identified organizational, financial, and community factors associated with reporting.

**Study design:**

Retrospective longitudinal observational study.

**Methods:**

We analyzed 13,876 hospital-year observations from 3358 nonfederal acute care hospitals between 2018 and 2022 using linked data from the American Hospital Association Annual Survey, the National Academy for State Health Policy Hospital Cost Report Tool, and the CDC/ATSDR Social Vulnerability Index (SVI). Population-averaged generalized estimating equation logistic regression models estimated associations between annual program reporting and hospital characteristics, including county socioeconomic vulnerability, with year fixed effects.

**Results:**

Across hospital-years, 66.8% reported a WVPP and 15.4% reported a CVPP. Nonprofit ownership, major teaching status, urban location, higher nurse staffing intensity, and larger hospital size were associated with higher odds of reporting both program types. Major teaching hospitals showed especially strong associations with WVPP (OR = 1.99, 95% CI 1.47–2.68) and CVPP (OR = 2.29, 95% CI 1.66–3.17) reporting. For WVPP, higher emergency department visits per bed were positively associated with reporting (OR = 1.25, 95% CI 1.14–1.36), whereas higher county socioeconomic vulnerability was negatively associated (OR = 0.62, 95% CI 0.48–0.80). County socioeconomic vulnerability was not significantly associated with CVPP reporting. Relative to 2018, the odds of reporting both programs increased over time.

**Conclusions:**

Violence prevention reporting increased over time but remained uneven across hospital types. WVPPs were far more common than CVPPs, and both were concentrated in hospitals with greater organizational capacity. Targeted policy and implementation support may be needed to expand prevention infrastructure in lower-resourced settings.

## 1 Introduction

Violence affecting hospitals exists along two interconnected fronts. Internally, workplace violence against healthcare workers has increased sharply: in 2023, 81.6% of nurses reported violence and more than half of emergency nurses experienced assault or threats within the prior month [1,2]. Externally, community violence creates downstream clinical and financial pressures, as up to 11% of violently injured patients return to the emergency department with a recurrent violent injury within three years [3]. These burdens also carry financial consequences. Hospitals absorb costs associated with workforce injury, turnover, and security infrastructure, while also bearing the clinical and social costs of treating violence-related injury, reinjury, and post-discharge resource needs. In 2023 alone, violence-related costs to U.S. hospitals exceeded $18 billion, including prevention infrastructure and post-event care [4]. Together, these internal and external pressures have pushed hospitals to respond both as employers responsible for workforce safety and as community partners confronting violence as a public health issue.

Accordingly, prevention efforts tend to fall into two corresponding domains aligned with these fronts: Workplace Violence Prevention Programs (WVPPs) and Community Violence Prevention Programs (CVPP). WVPPs focus on employee safety and are defined as multicomponent strategies integrating policies, reporting systems, environmental controls, and staff training to prevent aggression toward healthcare personnel [5]. Although accreditation and state requirements have increased the presence of WVPPs nationwide, particularly following the Joint Commission's 2022 workplace violence prevention standards [6], implementation remains uneven. For example, some systems (e.g., SSM Health) report increased staff preparedness through interdisciplinary team training, access control redesign, and the application of crime prevention through environmental design principles [7]. However, national studies indicate that many hospitals lack comprehensive program features, and adoption does not necessarily indicate depth, scope, or operational reach [8,9].

Community-focused efforts reflect hospitals’ expanding role as partners in local violence prevention ecosystems. The American Hospital Association defines community violence intervention as an evidence-informed approach to reduce violence by addressing social and economic conditions that contribute to harm and retaliation [10]. CVPPs operationalize this model by identifying violently injured patients at the point of care and providing trauma-informed case management, mental health support, social services navigation, and community partnership linkages [11,12]. Programs have shown success in helping victims achieve short-term goals focused on immediate basic needs, though evidence regarding long-term outcomes such as sustained reinjury reduction remains mixed [[13], [14], [15], [16], [17]]. While some individual programs have reported positive outcomes among motivated patients, systematic reviews have found insufficient high-quality evidence to make strong recommendations about adult-focused CVPPs [[18], [19], [20]].

Despite growing attention to hospital-based violence prevention, national research rarely examines WVPP and CVPP adoption together. Most prior studies focus on specific clinical settings (e.g., emergency departments), single program types, or limited geographic area, making it difficult to compare patterns across the hospital sector [21]. To address this gap, we used a national sample of U.S. acute care hospitals from 2018 to 2022 to (1) describe the prevalence and trends in WVPP and CVPP reporting and (2) examine the organizational, financial, and community factors associated with reported program presence. By considering both program types together, we sought to better understand how hospitals address violence as both a workforce safety challenge and a broader public health concern.

## 2 Methods

### 2.1 Study design and data sources

We conducted a retrospective longitudinal observational study of U.S. acute care hospitals using data from multiple national sources. Hospital characteristics and annual reporting of workplace violence prevention programs (WVPPs) and community violence prevention programs (CVPPs) were obtained from the American Hospital Association (AHA) Annual Survey, which collects data from more than 6000 U S. hospitals annually via voluntary survey, with a consistent response rate exceeding 75% [22]. The survey captures key domains including hospital demographics, organizational characteristics, and staffing. Hospital financial data were drawn from the National Academy for State Health Policy (NASHP) Hospital Cost Report Tool, which compiles information from Medicare cost reports [23]. County socioeconomic context was measured using the Social Vulnerability Index (SVI), a composite measure developed by the Centers for Disease Control and Prevention and Agency for Toxic Substances and Disease Registry (CDC/ATSDR) to capture community-level vulnerability to external stressors [24]. We used the 2020 SVI socioeconomic theme – which captures poverty, unemployment, lack of high school diploma, housing cost burden, expressed as a percentile ranking relative to all U.S. census tracts – as a time-invariant, county-level structural measure.

These data sources were merged at the hospital–year level using unique hospital identifiers and county FIPS codes, with AHA records linked to SVI data via five-digit county FIPS codes (match rate: 99.96%) and financial data merged using Medicare provider identifiers. The final analytic sample spanned 2018–2022; the 2023 data year was excluded due to incomplete violence prevention program reporting. Because the study relied exclusively on de-identified or publicly available data, it was classified as non-human subjects research.

### 2.2 Study sample

The analytic sample consisted of nonfederal acute care hospitals observed longitudinally from 2018 through 2022. Acute care hospitals were identified using AHA service code 10. Federal hospitals were excluded because mandatory federal violence prevention requirements limited meaningful variation in program reporting. Hospitals without emergency departments (VEM = 0) and hospitals with fewer than 25 beds were also excluded because they represent specialty facilities, freestanding emergency departments or micro-hospitals rather than general acute care facilities. After applying these restrictions, the analytic file contained 21,276 hospital-year observations representing 4406 unique hospitals.

### 2.3 Missing data

We examined patterns of missing data before multivariable analysis. Missingness was concentrated in the WVPP and CVPP variables, which were jointly missing for 5102 hospital-year observations, suggesting a common survey nonresponse mechanism. All remaining covariates were available for 99.96% of observations following SVI linkage. Outcome missingness was more common among rural and non-teaching hospitals, indicating that complete-case analyses may underrepresent less-resourced settings. No imputation procedures were performed. The final analytic sample for multivariable models using SVI specification included 13,876 hospital-year observations representing 3358 hospitals.

### 2.4 Outcome measures

The study included two binary outcomes derived from the AHA Annual Survey: whether a hospital reported having a workplace violence prevention program (WVPP) and whether it reported having a community violence prevention program (CVPP) in a given year. Each outcome was coded as 1 if the hospital reported the presence of the program and 0 otherwise. Because the unit of analysis was the hospital-year, these variables represent annual program reporting rather than one-time adoption.

### 2.5 Independent variables

Independent variables were selected based on prior literature on hospital safety, organizational capacity, and community violence prevention, and were grouped into organizational, financial, and community-level characteristics [11]. Organizational characteristics were derived from the AHA Annual Survey and included hospital ownership (for-profit, nonprofit, and nonfederal government, with for-profit as the reference category), system membership (system-affiliated vs. independent hospitals), teaching status (non-teaching, teaching, and major teaching), and urban–rural location (urban vs. rural). To capture workforce capacity, we included registered nurse full-time equivalents per staffed bed (RN FTEs per bed) as a standardized measure of staffing intensity. Emergency department (ED) utilization was measured as ED visits per staffed bed and log-transformed to address right skewness and to represent relative patient throughput and exposure to acute care demand.

Financial performance was assessed using operating profit margin obtained from the NASHP Hospital Cost Tool, reflecting hospitals' short-term financial flexibility and capacity to invest in prevention infrastructure. County-level socioeconomic context was measured using the Social Vulnerability Index (SVI) socioeconomic theme from CDC/ATSDR, treated as a time-invariant structural characteristic of the hospital's community context, as described above.

### 2.6 Statistical analysis

The primary unit of analysis was the hospital-year. We summarized hospital-year characteristics overall and by annual WVPP and CVPP reporting status. Categorical variables were compared using Pearson chi-square tests. Continuous variables with approximately symmetric distributions were compared using two-sample t-tests, whereas skewed continuous variables were compared using Wilcoxon rank-sum tests.

We estimated separate population-averaged generalized estimating equation (GEE) [25] logistic regression models for WVPP and CVPP, using robust standard errors and an exchangeable working correlation structure to account for repeated observations over time. Adjusted models included ownership, system membership, teaching status, urbanicity, registered nurse full-time equivalents per bed, log emergency department visits per bed, operating profit margin, hospital bed size, and the SVI socioeconomic theme. Year fixed effects were included in all models, with 2018 as the reference year. Results are reported as odds ratios (ORs) with 95% confidence intervals (CIs). Sensitivity analysis replaced the SVI measure with county homicide and unemployment variables Table 3). Where applicable, lagged versions of these county measures were also evaluated (Table 4). Additionally, we examined Medicaid payer mix as a hospital-level proxy for patient socioeconomic disadvantage (Table 5).

All analyses were performed using Stata/SE 17 (StataCorp LLC, College Station, TX), with statistical significance defined as a two-sided p < 0.05.

## 3 Results

The final analytic sample included 13,876 hospital-year observations from 3358 unique nonfederal acute care hospitals observed between 2018 and 2022. Across hospital-years, 9263 (66.8%) reported a WVPP and 2132 (15.4%) reported a CVPP, indicating that workplace-focused programs were substantially more common than community-focused programs.

### 3.1 Descriptive characteristics

#### 3.1.1 Workplace violence prevention program reporting

As shown in Table 1, Panel A, hospital-years reporting a WVPP differed significantly from non-reporting hospital-years across all major organizational characteristics. WVPP reporting was more common among non-profit hospitals, which accounted for 74.5% of WVPP-reporting hospital-years compared with 61.7% of non-reporting hospital-years, whereas for-profit hospitals were underrepresented among WVPP reporters (10.0% vs 14.3%). System membership was also more common among WVPP-reporting hospital-years (74.9% vs 66.0%). A clear gradient was observed by teaching intensity: major teaching hospitals comprised 10.3% of WVPP-reporting hospital-years but only 2.2% of non-reporting hospital-years. Similarly, WVPP reporting was substantially more common among urban hospitals (72.6% vs 49.1%) and larger hospitals, with facilities containing 300 or more beds accounting for 29.0% of reporting hospital-years compared with 11.4% of non-reporting hospital-years. Hospital-years reporting a WVPP also had higher mean nurse staffing intensity (1.62 vs 1.31 RN full-time equivalents per bed, p < 0.001), higher median operating profit margins (0.153 vs 0.119, p < 0.001), and slightly lower mean SVI socioeconomic scores (0.506 vs 0.530, p < 0.001).

**Table 1:** Characteristics of nonfederal U.S. Hospital-Years by violence prevention Program reporting, 2018–2022.

Panel A. Workplace Violence Prevention Program (WVPP), n = 13,876			
Characteristic	No WVPP reported (n = 4613)	WVPP reported (n = 9263)	p-value
Organizational characteristics, n (%)			
**Ownership**			<0.001
For-profit	660 (14.3)	930 (10.0)	
Nonprofit	2845 (61.7)	6905 (74.5)	
Government, nonfederal	1108 (24.0)	1428 (15.4)	
**System membership**			<0.001
Non-system hospital	1569 (34.0)	2324 (25.1)	
System member	3045 (66.0)	6939 (74.9)	
**Teaching status**			<0.001
Non-teaching	3363 (72.9)	4771 (51.5)	
Teaching	1150 (24.9)	3534 (38.1)	
Major teaching	101 (2.2)	958 (10.3)	
**Location**			<0.001
Rural	2348 (50.9)	2541 (27.4)	
Urban	2266 (49.1)	6722 (72.6)	
**Hospital bed size**			<0.001
<100 beds	2718 (58.9)	3073 (33.2)	
100–299 beds	1368 (29.6)	3502 (37.8)	
≥300 beds	528 (11.4)	2688 (29.0)	
**Capacity and operational characteristics**			
RN full-time equivalents per bed, mean (SD)	1.31 (0.73)	1.62 (0.78)	<0.001
Log emergency department visits per bed, mean (SD)	5.13 (0.88)	5.26 (0.64)	0.161
**Financial performance**			
Operating profit margin, median (IQR)	0.119 (−0.002 to 0.217)	0.153 (0.061 to 0.228)	<0.001
**Community context**			
SVI socioeconomic theme, mean (SD)	0.530 (0.280)	0.506 (0.277)	<0.001

Panel B. Community Violence Prevention Program (CVPP)			
Characteristic	No CVPP reported (n = 11,744)	CVPP reported (n = 2132)	p-value
Organizational characteristics, n (%)			
**Ownership**			<0.001
For-profit	1485 (12.6)	105 (4.9)	
Nonprofit	7998 (68.1)	1752 (82.2)	
Government, nonfederal	2261 (19.3)	275 (12.9)	
**System membership**			<0.001
Non-system hospital	3445 (29.3)	448 (21.0)	
System member	8300 (70.7)	1684 (79.0)	
**Teaching status**			<0.001
Non-teaching	7442 (63.4)	692 (32.5)	
Teaching	3815 (32.5)	869 (40.8)	
Major teaching	488 (4.2)	571 (26.8)	
**Location**			<0.001
Rural	4578 (39.0)	311 (14.6)	
Urban	7167 (61.0)	1821 (85.4)	
**Hospital bed size**			<0.001
<100 beds	5385 (45.9)	406 (19.0)	
100–299 beds	4243 (36.1)	627 (29.4)	
≥300 beds	2117 (18.0)	1099 (51.5)	
**Capacity and operational characteristics**			
RN full-time equivalents per bed, mean (SD)	1.45 (0.75)	1.88 (0.82)	<0.001
Log emergency department visits per bed, mean (SD)	5.23 (0.76)	5.15 (0.56)	<0.001
**Financial performance**			
Operating profit margin, median (IQR)	0.138 (0.035 to 0.221)	0.167 (0.084 to 0.245)	<0.001
**Community context**			
SVI socioeconomic theme, mean (SD)	0.517 (0.277)	0.496 (0.281)	0.002

**Notes.** Unit of analysis is the hospital-year. Values are reported as n (%) for categorical variables, mean (SD) for approximately normally distributed continuous variables, and median (IQR) for skewed continuous variables. P-values are from Pearson chi-square tests for categorical variables, two-sample t tests for normally distributed continuous variables, and Wilcoxon rank-sum tests for skewed continuous variables. Descriptive comparisons are unadjusted.

#### 3.1.2 Community violence prevention program reporting

Table 1, Panel B shows that CVPP reporting was considerably less common than WVPP reporting and was more highly concentrated among specific hospital types. Nonprofit hospitals accounted for 82.2% of CVPP-reporting hospital-years compared with 68.1% of non-reporting hospital-years, whereas for-profit hospitals represented only 4.9% of reporters despite comprising 12.6% of non-reporters. This ownership pattern was more pronounced for CVPP than for WVPP. Teaching intensity showed a similarly strong pattern: major teaching hospitals comprised 26.8% of CVPP-reporting hospital-years but only 4.2% of non-reporting hospital-years. Urban location was also strongly associated with CVPP reporting (85.4% vs 61.0%). Hospitals reporting CVPPs were substantially larger, with 51.5% having 300 or more beds compared with 18.0% of non-reporting hospital-years. In addition, CVPP-reporting hospital-years had higher mean nurse staffing intensity (1.88 vs 1.45 RN full-time equivalents per bed, p < 0.001), higher median operating profit margins (0.167 vs 0.138, p < 0.001), and slightly lower mean SVI socioeconomic scores (0.496 vs 0.517, p = 0.002). Notably, log emergency department visit volume was slightly lower among hospital-years reporting CVPPs than among non-reporting hospital-years (5.15 vs 5.23, p < 0.001), a pattern that differed from WVPP reporting and was examined further in multivariable analyses.

### 3.2 Multivariable analyses results

Table 2 presents population-averaged GEE logistic regression models estimating the odds that a hospital reported a WVPP or CVPP in a given year. In both models, nonprofit and nonfederal government hospitals had higher odds of reporting violence prevention programs than for-profit hospitals. Major teaching hospitals, urban hospitals, hospitals with higher RN staffing intensity, and hospitals with 300 or more beds also had higher odds of reporting both program types.

**Table 2:** Population-averaged GEE logistic regression models of annual violence prevention program reporting, 2018–2022.

Variable	WVPP		CVPP	
	OR (95% CI)	p	OR (95% CI)	p
Ownership (ref: For-profit)				
Nonprofit	1.64 (1.28–2.10)	<0.001	2.89 (1.88–4.43)	<0.001
Government, nonfederal	1.51 (1.06–2.15)	0.023	1.95 (1.25–3.06)	0.003
System member	1.16 (1.00–1.34)	0.052	0.91 (0.76–1.11)	0.362
**Teaching (ref: Non-teaching)**				
Teaching	1.13 (1.01–1.26)	0.031	1.13 (0.99–1.30)	0.079
Major teaching	1.99 (1.47–2.68)	<0.001	2.29 (1.66–3.17)	<0.001
Urban (ref: Rural)	1.50 (1.26–1.78)	<0.001	1.96 (1.45–2.66)	<0.001
RN FTE per bed	1.10 (1.02–1.17)	0.009	1.17 (1.07–1.28)	0.001
Log ED visits per bed	1.25 (1.14–1.36)	<0.001	0.94 (0.84–1.05)	0.275
Operating profit margin	1.20 (1.00–1.45)	0.054	1.20 (0.94–1.52)	0.139
**Bed size (ref: <100 beds)**				
100–299 beds	1.64 (1.37–1.97)	<0.001	1.26 (0.91–1.74)	0.173
≥300 beds	2.21 (1.76–2.77)	<0.001	2.05 (1.46–2.89)	<0.001
SVI socioeconomic theme	0.62 (0.48–0.80)	<0.001	0.72 (0.47–1.09)	0.123
**Year (ref: 2018)**				
2019	1.10 (1.06–1.14)	<0.001	1.04 (0.99–1.09)	0.086
2020	1.24 (1.18–1.31)	<0.001	1.13 (1.05–1.22)	0.001
2021	1.34 (1.26–1.42)	<0.001	1.17 (1.07–1.27)	0.001
2022	1.56 (1.45–1.68)	<0.001	1.24 (1.13–1.35)	<0.001
Observations	13,876		13,876	
Hospitals	3358		3358	
Wald χ^2^	476.28	<0.001	315.49	<0.001

**Notes.** Values are odds ratios (ORs) with 95% confidence intervals from population-averaged generalized estimating equation (GEE) logistic regression models with robust standard errors and an exchangeable working correlation structure. The unit of analysis is the hospital-year. Models include year fixed effects and are estimated on the complete-case analytic sample of nonfederal acute care hospitals from 2018 to 2022. Reference categories are for-profit ownership, non-teaching status, rural location, and bed size <100 beds.

For WVPP, nonprofit ownership (OR, 1.64; 95% CI, 1.28–2.10), government nonfederal ownership (OR, 1.51; 95% CI, 1.06–2.15), teaching status (OR, 1.13; 95% CI, 1.01–1.26), major teaching status (OR, 1.99; 95% CI, 1.47–2.68), urban location (OR, 1.50; 95% CI, 1.26–1.78), higher RN staffing per bed (OR, 1.10; 95% CI, 1.02–1.17), and larger bed size were all associated with higher odds of reporting a program. Hospitals with 100–299 beds had 1.64 times the odds of reporting WVPP relative to hospitals with fewer than 100 beds, and hospitals with 300 or more beds had 2.21 times the odds. Higher emergency department visits per bed were also positively associated with WVPP reporting (OR, 1.25; 95% CI, 1.14–1.36). In contrast, the SVI socioeconomic theme was negatively associated with WVPP reporting (OR, 0.62; 95% CI, 0.48–0.80), indicating lower odds of reported WVPP in more socioeconomically vulnerable counties.

For CVPP, nonprofit ownership (OR, 2.89; 95% CI, 1.88–4.43), government nonfederal ownership (OR, 1.95; 95% CI, 1.25–3.06), major teaching status (OR, 2.29; 95% CI, 1.66–3.17), urban location (OR, 1.96; 95% CI, 1.45–2.66), higher RN staffing per bed (OR, 1.17; 95% CI, 1.07–1.28), and bed size of 300 or more beds (OR, 2.05; 95% CI, 1.46–2.89) were associated with higher odds of reporting a program. Mid-sized hospitals (100–299 beds) did not differ significantly from hospitals with fewer than 100 beds, and neither system membership, log emergency department visits per bed, operating profit margin, nor the SVI socioeconomic theme was significantly associated with CVPP reporting. Teaching hospitals showed a positive but non-significant association with CVPP reporting (OR, 1.13; 95% CI, 0.99–1.30).

Relative to 2018, the odds of reporting WVPP increased in every subsequent year, with the largest increase observed in 2022 (OR, 1.56; 95% CI, 1.45–1.68). For CVPP, year effects were smaller and less consistent: 2019 was not significantly different from 2018, but the odds of CVPP reporting were significantly higher in 2020, 2021, and 2022.

## 4 Discussion

This national hospital-year analysis shows that reporting of WVPP and CVPP is associated with organizational characteristics, with a more limited and differentiated role for community context. WVPPs were substantially more common than CVPPs, a pattern consistent with hospitals directing resources toward workforce safety initiatives, often driven by regulatory standards and accreditation expectations [26,27]. Across 2018-2022, the odds of reporting both program types increased over time, although WVPP reporting remained considerably more prevalent than CVPP reporting. Together, these findings reveal an uneven distribution of violence prevention infrastructure across hospitals, concentrated in settings with greater organizational capacity. According to the literature, key barriers to broader adoption include lack of funding, inadequate program staffing and resources, difficulty recruiting and retaining patients from under-resourced communities, and limited clinician awareness of available services [21,28].

Ownership was one of the most consistent correlates of program reporting. Nonprofit and nonfederal government hospitals had higher odds of reporting both WVPPs and CVPPs than for-profit hospitals, with especially large differences for CVPPs. This pattern is consistent with the possibility that mission orientation – reflecting the community benefit obligations and anchor institution roles that characterize nonprofit and public hospitals [29] – may facilitate investments in violence prevention that extend beyond direct clinical care. Nonprofit hospitals, which receive tax-exempt status in exchange for providing community benefit, operate under both regulatory expectations and informal institutional commitments to address the upstream drivers of health in their surrounding communities, and violence prevention may represent one expression of that orientation [30]. In contrast, for-profit hospitals may face stronger pressure to prioritize activities with more immediate financial returns, which may make sustained investment in prevention programming less likely. These ownership differences underscore the need for targeted policy or financing mechanisms to support violence prevention implementation in hospitals with fewer mission-driven incentives.

Teaching status also emerged as a strong predictor, particularly for major teaching hospitals. Hospitals with major teaching status had substantially higher odds of reporting both WVPPs and CVPPs, even after adjustment for ownership, urbanicity, staffing, financial performance, and bed size. The pattern likely reflects several reinforcing mechanisms: larger institutional scale and resource intensity, which are more common in teaching hospitals than in community hospitals [31], greater access to interdisciplinary expertise [32,33], stronger ties to academic and public health partners [34], and higher exposure to violence-related injuries through trauma services [35]. Teaching hospitals may also face greater regulatory scrutiny and reputational incentives to adopt formalized violence prevention strategies [36].

Hospital size and staffing capacity were also important correlates of program reporting. Larger hospitals, particularly those with 300 or more beds, were more likely to report both program types, and higher RN staffing intensity was positively associated with both WVPP and CVPP reporting. These findings support the interpretation that violence prevention infrastructure depends in part on organizational capacity, i.e., hospitals with higher RN staffing may have a greater ability to absorb the operational costs of program development, training, and coordination [37]. However, the mechanism linking staffing intensity to violence prevention program reporting is likely more complex than resource availability alone. Earlier work suggested that a pathway from poor staffing through increased workload to burnout and physical injury among nurses [38], implying that well-staffed hospitals may adopt WVPPs partly to interrupt this chain. Yet more recent evidence from acute mental health settings found no consistent association between nurse staffing levels and violence-related outcomes [39], suggesting this relationship may not generalize uniformly across care contexts. An alternative and complementary explanation is that financially healthy, well-staffed hospitals may be more likely to comply with Joint Commission standards encouraging the formation of workplace safety committees [40,41]. Staffing intensity may therefore reflect not only operational capacity but also broader organizational investments in workplace safety and well-being.

Urban location was another consistent correlate of both WVPP and CVPP reporting. Urban hospitals had substantially higher odds of reporting both types of programs, which may reflect both greater exposure to violence-related clinical and operational demands [42] and greater access to the external networks often required for effective prevention and referral-based interventions [32,43]. This urban concentration is notable because it raises concern that hospitals serving rural communities may have less access to the resources, partnerships, and implementation capacity needed to establish formal prevention infrastructure.

The role of ED volume differed across program types. Greater ED visits per bed were positively associated with WVPP reporting but were not significantly associated with CVPP reporting in the fully adjusted models. This pattern reflects the close link between high-intensity front-line care environments and internal workforce violence prevention, where elevated exposure to acute behavioral and safety challenges creates direct operational pressure for program adoption. By contrast, CVPP reporting appears less tightly linked to ED volume once other organizational characteristics are taken into account, suggesting that community violence programming may depend more on institutional mission and capacity than on ED throughput alone.

Community socioeconomic vulnerability showed a more limited and program-specific association with violence prevention reporting. Higher SVI socioeconomic vulnerability was associated with lower odds of WVPP reporting, whereas it was not significantly associated with CVPP reporting. This pattern points to a potential inverse relationship between community disadvantage and formal workplace violence prevention infrastructure – one that persists after accounting for hospital ownership, teaching status, staffing, size, and urbanicity. A plausible explanation is that hospitals serving more socioeconomically vulnerable communities face greater resource constraints that impede the formalization of prevention programs, even in contexts where need may be elevated. The asymmetry between program types is notable: the absence of a significant SVI association for CVPP reporting might indicate that community-facing prevention efforts are shaped more by institutional mission, hospital size, and teaching intensity than by county-level socioeconomic context. Why resource constraints would differently suppress WVPP but not CVPP adoption remains an open question and warrants further investigation.

Sensitivity analyses provided additional context for interpreting the primary findings. Models using county homicide rate and unemployment showed generally similar organizational patterns but were accompanied by substantially greater and more selective missingness, with underrepresentation of rural, government, and less-resourced hospitals (Table 3). For that reason, the SVI-based models provide a more stable primary specification for the national hospital-year sample. Additional analyses incorporating Medicaid payer mix (Table 4) suggested that hospitals serving larger low-income patient populations were more likely to report violence prevention programs, supporting the idea that patient socioeconomic burden is relevant to implementation, although this measure captures hospital patient mix rather than county structural vulnerability.

**Table 3:** Sensitivity analysis using county homicide rate and unemployment rate.

Predictor	WVPP OR (95% CI)	p	CVPP OR (95% CI)	p
Ownership (ref: For-profit)				
Nonprofit	2.03 (1.57, 2.62)	<0.001	3.93 (2.62, 5.90)	<0.001
Gov nonfederal	1.93 (1.26, 2.97)	0.003	2.39 (1.52, 3.76)	<0.001
**System member (ref: No)**				
Yes	1.30 (1.08, 1.57)	0.005	0.87 (0.70, 1.09)	0.220
**Teaching status (ref: Nonteaching)**				
Teaching	1.19 (1.07, 1.33)	0.001	1.25 (1.09, 1.44)	0.002
Major teaching	2.32 (1.76, 3.06)	<0.001	2.76 (1.98, 3.84)	<0.001
**Urban location (ref: Rural)**				
Urban	1.69 (1.38, 2.06)	<0.001	2.13 (1.44, 3.13)	<0.001
RN FTEs per bed	1.01 (0.95, 1.09)	0.673	1.11 (1.06, 1.18)	<0.001
ln ED visits per bed	1.02 (0.94, 1.12)	0.634	0.86 (0.78, 0.95)	0.002
Operating profit margin	1.27 (0.94, 1.70)	0.116	1.50 (1.04, 2.18)	0.031
County homicide rate	0.98 (0.97, 0.99)	0.004	1.01 (0.99, 1.03)	0.270
County unemployment rate	1.13 (1.07, 1.19)	<0.001	1.10 (1.04, 1.17)	0.001
**Year (ref: 2018)**				
2019	1.10 (1.05, 1.14)	<0.001	1.05 (1.00, 1.10)	0.063
2020	1.21 (1.14, 1.28)	<0.001	1.14 (1.05, 1.23)	0.001
2021	1.30 (1.21, 1.39)	<0.001	1.17 (1.07, 1.28)	0.001
2022	1.58 (1.45, 1.72)	<0.001	1.27 (1.15, 1.40)	<0.001

Notes: Odds ratios (ORs) and 95% confidence intervals are from GEE logistic models with exchangeable correlation structure and robust standard errors clustered by hospital. Analytic sample: 10,906 hospital-years from 2587 hospitals. Reference categories: for-profit ownership, non-system hospitals, nonteaching hospitals, and rural location. Year reference = 2018.

**Table 4:** Sensitivity analysis using one-year lagged county contextual measures Lagged county homicide rate and lagged county unemployment rate.

Predictor	WVPP OR (95% CI)	p	CVPP OR (95% CI)	p
Ownership (ref: For-profit)				
Nonprofit	1.91 (1.47, 2.48)	<0.001	3.96 (2.56, 6.14)	<0.001
Gov nonfederal	1.73 (1.13, 2.65)	0.011	2.50 (1.52, 4.09)	<0.001
**System member (ref: No)**				
Yes	1.44 (1.18, 1.75)	<0.001	0.91 (0.72, 1.16)	0.465
**Teaching status (ref: Nonteaching)**				
Teaching	1.33 (1.15, 1.53)	<0.001	1.45 (1.21, 1.74)	<0.001
Major teaching	2.77 (2.05, 3.75)	<0.001	3.07 (2.13, 4.42)	<0.001
**Urban location (ref: Rural)**				
Urban	1.50 (1.22, 1.84)	<0.001	1.97 (1.33, 2.93)	0.001
RN FTEs per bed	1.04 (0.96, 1.13)	0.298	1.15 (1.05, 1.26)	0.002
ln ED visits per bed	1.03 (0.94, 1.13)	0.480	0.84 (0.76, 0.94)	0.002
Operating profit margin	1.09 (0.80, 1.49)	0.580	1.29 (0.89, 1.85)	0.175
Lagged county homicide rate	0.98 (0.96, 0.99)	<0.001	1.01 (0.99, 1.03)	0.236
Lagged county unemployment rate	1.12 (1.05, 1.18)	<0.001	1.09 (1.03, 1.16)	0.004
**Year (ref: 2019)**				
2020	1.10 (1.05, 1.15)	<0.001	1.07 (1.01, 1.14)	0.035
2021	1.18 (1.11, 1.25)	<0.001	1.12 (1.03, 1.21)	0.007
2022	1.43 (1.33, 1.55)	<0.001	1.20 (1.10, 1.32)	<0.001

**Table 5:** Sensitivity analysis adding Medicaid payer mix Lagged county-context models additionally adjusted for Medicaid payer mix.

Predictor	WVPP OR (95% CI)	p	CVPP OR (95% CI)	p
Ownership (ref: For-profit)				
Nonprofit	1.93 (1.48, 2.51)	<0.001	3.95 (2.53, 6.18)	<0.001
Gov nonfederal	1.63 (1.06, 2.52)	0.027	2.32 (1.40, 3.84)	0.001
**System member (ref: No)**				
Yes	1.31 (1.10, 1.57)	0.003	0.91 (0.72, 1.16)	0.445
**Teaching status (ref: Nonteaching)**				
Teaching	1.29 (1.12, 1.49)	0.001	1.44 (1.20, 1.74)	<0.001
Major teaching	2.68 (1.97, 3.64)	<0.001	3.27 (2.30, 4.65)	<0.001
**Urban location (ref: Rural)**				
Urban	1.55 (1.27, 1.91)	<0.001	2.01 (1.34, 3.01)	0.001
RN FTEs per bed	1.03 (0.96, 1.12)	0.395	1.16 (1.06, 1.27)	0.001
ln ED visits per bed	1.03 (0.94, 1.13)	0.573	0.84 (0.75, 0.94)	0.002
Operating profit margin	1.08 (0.79, 1.47)	0.650	1.36 (0.93, 1.97)	0.111
Medicaid payer mix (%)	1.01 (1.00, 1.02)	0.009	1.01 (1.00, 1.02)	0.003
Lagged county homicide rate	0.98 (0.96, 0.99)	0.001	1.01 (0.99, 1.03)	0.248
Lagged county unemployment rate	1.08 (1.02, 1.15)	0.010	1.05 (0.99, 1.12)	0.122
**Year (ref: 2019)**				
2020	1.10 (1.05, 1.15)	<0.001	1.07 (1.00, 1.14)	0.044
2021	1.18 (1.11, 1.25)	<0.001	1.11 (1.02, 1.20)	0.013
2022	1.42 (1.31, 1.53)	<0.001	1.18 (1.07, 1.29)	0.001

Notes: Same specification as Table 2, with Medicaid payer mix added as a hospital-level proxy for patient socioeconomic disadvantage/safety-net burden. Analytic sample: 8375 hospital-years from 2455 hospitals. Medicaid payer mix is modeled per 1 percentage-point increase. Year reference = 2019.

Several implications follow from these findings. First, violence prevention reporting appears to be concentrated in hospitals with greater institutional capacity, especially large, urban, nonprofit, and major teaching hospitals. Second, the markedly lower prevalence of CVPP reporting suggests that community-facing prevention remains substantially less established than internal workplace prevention. Third, hospitals located in more socioeconomically vulnerable areas may be less likely to report WVPPs, raising concern that the hospitals facing the greatest structural disadvantage may have the least formal prevention infrastructure. Taken together, these patterns suggest that voluntary diffusion alone may not produce equitable implementation of violence prevention programs. Smaller, rural, for-profit, and lower-capacity hospitals may require targeted technical assistance, cross-sector partnership support, and dedicated financing to implement and sustain effective prevention strategies.

### 4.1 Limitations

This study has several limitations. First, the AHA measures capture self-reported program presence and do not provide information on program intensity, fidelity, staffing, or effectiveness. Second, because the unit of analysis was the hospital-year, the models estimate associations with annual program reporting rather than the timing or determinants of first adoption. Third, although the primary SVI specification improved county-level coverage relative to models using homicide data, complete-case analysis may still underrepresent some less-resourced hospitals, particularly rural and non-teaching settings. Fourth, the SVI socioeconomic theme was treated as a time-invariant county characteristic and therefore reflects structural community context rather than annual short-term change. Furthermore, county-level measures may mask important within-county variation in violence burden, deprivation, and service access. Finally, the analysis did not fully differentiate pediatric and other specialty acute care hospitals from general community hospitals, nor did it directly model trauma designation, market competition, hospital density, or regional differences. These factors may influence both hospitals’ need for violence prevention programs and their capacity to implement them.

Future research should examine the quality, intensity, and sustainability of hospital violence prevention efforts; distinguish more clearly between program presence and implementation depth; and assess downstream effects on workforce safety, patient outcomes, reinjury, and community partnerships. Mixed-methods work may be especially useful for understanding why some hospitals, particularly those with fewer resources, do not establish or sustain formal violence prevention programs.

### 4.2 Conclusion

Reporting of violence prevention programs increased over time, but substantial gaps remain across hospital types. WVPPs were much more commonly reported than CVPPs, and both program types were concentrated in hospitals with greater organizational capacity, especially nonprofit, major teaching, urban, and larger hospitals. These findings suggest that the presence of hospital violence prevention infrastructure is shaped not only by recognition of violence as a workforce and public health issue, but also by hospitals’ capacity to translate that recognition into formal programming. Continued progress will likely depend on sustained policy attention, clearer implementation expectations, and targeted support for hospitals that currently have the fewest resources to build and maintain prevention infrastructure.

## Ethical statement

Not applicable; this study does not constitute human subject research.

## Declaration of generative AI and AI-assisted technologies in the manuscript preparation process

During the preparation of this work, the author(s) used ChatGPT for outlining, restructuring sentences, checking flow and logic, refining the English language, and proofreading purposes. After using this tool, the author(s) reviewed and edited the content as needed and take full responsibility for the content of the published article.

## Funding

No external funding was received for this study.

## Conflict of interest

We, the undersigned authors, hereby declare that we have no conflicts of interest to disclose in relation to this manuscript.

No author has any financial or non-financial competing interests that could inappropriately influence, or be perceived to influence, the work reported in this manuscript.

## Data Availability

Data were accessed through institutional licensing and use agreements; raw data cannot be publicly shared per licensing terms.
